# Defined Dinuclear Nickel Sites on Mesoporous Silica Translate Molecular Surface Mimics to Heterogeneous Catalysis

**DOI:** 10.1002/anie.5073615

**Published:** 2026-08-23

**Authors:** Jitpisut Poolwong, Adrian Jenner, Eric Juriatti, Mustafa Turan, Manfred Manßen

**Affiliations:** ^1^ Institut Für Anorganische Chemie Eberhard Karls Universität Tübingen (EKUT) Tübingen Germany; ^2^ Institut Für Physikalische und Theoretische Chemie Eberhard Karls Universität Tübingen (EKUT) Tübingen Germany; ^3^ Institut Für Technische Chemie Universität Stuttgart Stuttgart Germany

**Keywords:** cooperative catalysis, dinickel catalysis, dinuclear nickel sites, heterogeneous catalysis, surface immobilization

## Abstract

Metal–metal cooperativity in dinuclear transition‐metal systems offers unique opportunities for controlling reactivity and selectivity in catalytic transformations. Here, we report, inspired by molecular chemistry, the assembly of a bimetallic nickel system grafted onto mesoporous SBA‐15 (Ni‐NDI‐APTES@SBA‐15_250_). The well‐defined mesostructure of SBA‐15 enables the immobilization of dinuclear nickel units while preserving their proximity and cooperative reactivity. Comprehensive characterization, including PXRD, N_2_ physisorption, DRIFT‐IR, S/TEM, XPS, EA, ICP‐OES, TGA, and solid‐state NMR, confirms the successful incorporation of Ni_2_ species and retention of the mesoporous framework. The catalytic performance of Ni‐NDI‐APTES@SBA‐15_250_ was evaluated in the cyclotrimerization of terminal alkynes and compared with representative homogeneous mono‐ and dinuclear nickel catalysts. Ni‐NDI‐APTES@SBA‐15_250_ preserves the cooperative reactivity and high regioselectivity of the molecular dinuclear catalyst while the surface‐bound environment introduces a distinct, substrate‐dependent chemoselectivity, as exemplified by the selective monocyclotrimerization of diyne substrates instead of the double cyclization favored by the homogeneous analogue. Moreover, Ni‐NDI‐APTES@SBA‐15_250_ combines excellent catalytic performance with operational stability, recyclability, and negligible nickel leaching demonstrating that an immobilized catalyst can preserve molecular bimetallic reactivity while modulating catalytic pathways through the local supported environment.

## Graphical abstract

The cooperative reactivity characteristic of molecular dinuclear nickel complexes is transferred to mesoporous silica through a systematic immobilization strategy. The resulting material preserves the key features of the bimetallic motif within a robust heterogeneous framework, demonstrating how molecular design principles can be translated into well‐defined, reusable surface‐bound catalysts.

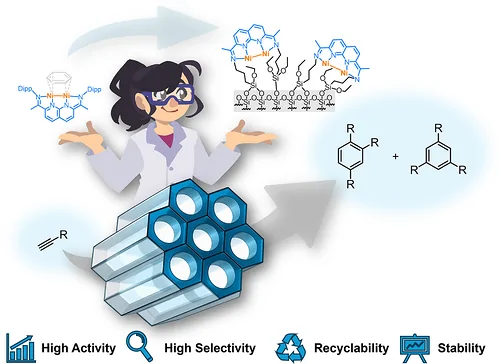

## 1 Introduction

Over the past decade, multimetallic catalysis has attracted significant attention because metal–metal (M–M) interactions can enable unique reactivities that mononuclear centers cannot access [1, 2, 3, 4, 5, 6, 7, 8, 9, 10]. The ability of two adjacent metals to share electrons, activate substrates simultaneously, or facilitate multistep bond‐forming events has driven extensive efforts in ligand design. The ligands require a suitable rigid framework to enforce precise M–M distances and promote electronic interactions [2, 11, 12, 13]. Moreover, ligand structures are not only essential for stabilizing M–M units but also for facilitating concerted catalytic pathways [2, 5, 6, 14, 15].

A closely related motivation comes from heterogeneous catalysis. Many industrial transformations are generally understood to proceed through cooperative activation across multiple metal atoms on a surface, and this has inspired the design of molecular multimetallic complexes as mimics of metallic surfaces that allow mechanistic interrogation [16, 17, 18, 19, 20, 21, 22, 23, 24, 25, 26, 27, 28]. Discrete bimetallic and cluster complexes can be engineered to reproduce key electronic and geometric aspects of surface ensembles, thereby testing whether a specific multinuclear motif is sufficient to reproduce a given cooperative reactivity [2, 22, 24, 25, 27, 28, 29, 30].

However, the reverse direction, transferring a molecularly defined cooperative motif back onto a solid surface while preserving its designed structure, remains far less developed [24, 28, 31, 32, 33, 34]. Even within surface organometallic chemistry (SOMC), where tailored molecular precursors are used to generate well‐defined supported species, the nature of the resulting surface sites can depend strongly on the preparation route, and multiple site types may coexist [16, 21, 27, 31, 35, 36, 37, 38, 39]. This highlights both the importance of synthetic methodology and the practical difficulty of obtaining truly bimetallic surface species.

Recently, dinucleating frameworks with nickel as the central element have been utilized in several unique catalytic reactions [1, 40, 41, 42, 43, 44, 45, 46, 47, 48]. For example, Meyer and coworkers have shown that pyrazolate‐bridged binucleating ligands can support dinuclear nickel complexes capable of cooperative small‐molecule activation (Scheme 1) [42, 49, 50, 51]. Similarly, Agapie and coworkers have developed dinuclear nickel architectures that enable unusual reactivity through closely spaced metal centers and ligand‐supported cooperativity [43, 45]. Among these ligand systems, naphthyridine diimines (NDIs) have become a prominent ligand platform for dinuclear chemistry, due to the rigidity of the naphthyridine backbone combined with conjugated imine groups that provide electronic flexibility and redox non‐innocence [52, 53, 54, 55, 56, 57]. The group of Uyeda investigated this ligand class to explore cooperative reactivity at dinuclear nickel sites (Scheme 1). Their studies showed that the redox‐active NDI ligand was involved in the redox process, stabilizing Ni(I)–Ni(I) metal complexes and enabling access to multielectron pathways that were not observable in mononuclear nickel species [44, 47, 48, 58, 59]. Moreover, mechanistic and computational studies have demonstrated that the dinuclear system of Ni(I)–Ni(I) centers leads to unique dinuclear intermediates that support its distinctive catalytic chemistry [1, 44, 46, 47, 48, 58, 59, 60].

**SCHEME 1:**
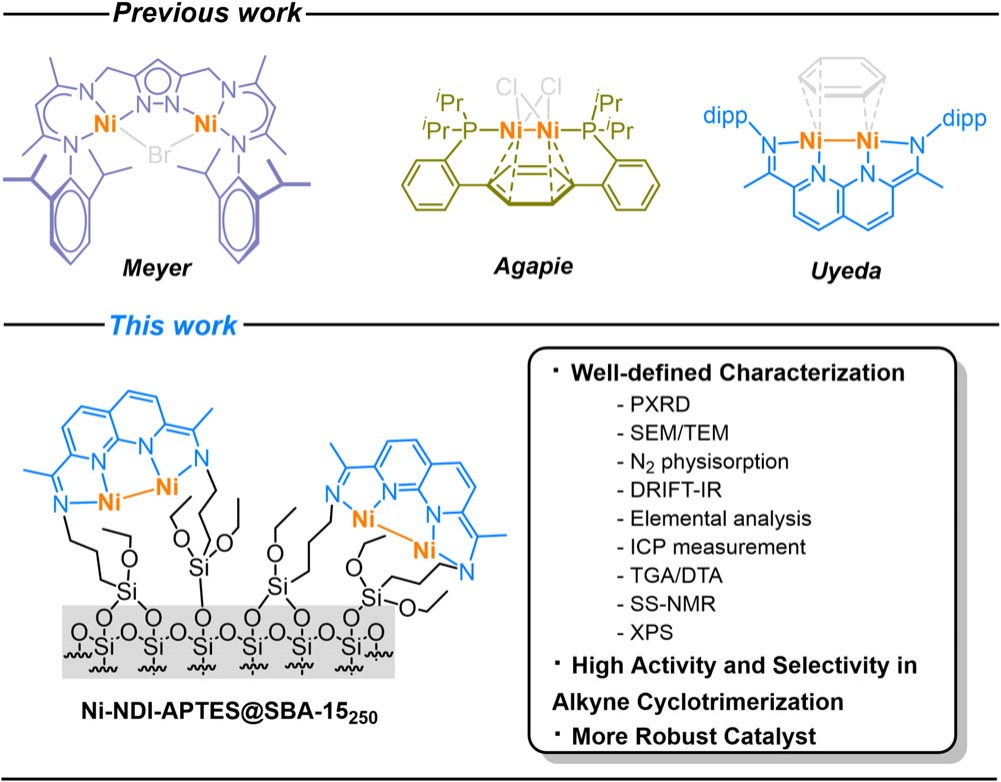
Selected Ni_2_ complexes in previous works [42, 43, 44] and the grafted material in this work.

Inspired by these mechanistic insights on the molecular level of dinuclear Ni(I)–Ni(I) complexes [44, 47, 48, 58, 59], we were interested in extending this concept to a heterogeneous system to explicitly test and realize the “surface cut‐out” idea in the reverse direction. We ask whether a molecularly designed, cooperative dinickel motif is not only a useful mimic of surface cooperativity, but can also be reimplemented as a defined bimetallic site on a solid support. Herein, we describe the first systematic synthesis of dinuclear Ni_2_‐NDI units (with a short metal–metal distance) [44, 58] on high‐surface‐area mesoporous silica, SBA‐15, through controlled surface functionalization and anchoring strategies. The SBA‐15 provides an ideal framework for immobilizing the Ni_2_‐NDI moiety while maintaining accessibility and structural stability, thanks to its tunable pore structure and thick walls [61, 62, 63, 64, 65, 66]. This approach enables precise control over the active site structure and offers valuable insights into the behavior of surface‐grafted active species.

To confirm the successful synthesis of the functionalized Ni_2_‐NDI heterogeneous materials, various comprehensive characterization techniques are employed, including powder XRD, N_2_ analysis, DRIFT‐IR, SEM, TEM, XPS, EA, ICP‐OES, TGA, and solid‐state NMR. All these combined characterizations confirm the successful immobilization and the presence of Ni_2_‐NDI on the silica surface. Finally, we evaluate the performance and recyclability of the heterogeneous material in terminal alkyne cyclotrimerization, showcasing specific regioselectivity consistent with the cooperative molecular Ni–Ni pathway [46].

## 2 Results and Discussion

### 2.1 Synthesis and Characterization

The periodic mesoporous silica (SBA‐15) was synthesized using a sol‐gel method, with tetraethyl orthosilicate (TEOS) as the silica precursor [67, 68]. The structural order and textural properties of the materials were confirmed by PXRD analysis and N_2_ physisorption, while SEM and TEM analyses revealed a rice‐shaped morphology, consistent with previous reports (Supporting Information, Figures S1 and S2) [67, 68].

Scheme 2 illustrates the stepwise synthetic route to the Ni‐NDI‐APTES@SBA‐15_250_ heterogeneous material. First, an amino‐functionalized silica surface (APTES@SBA‐15_250_) was prepared by reacting SBA‐15_250_ with 3‐aminopropyltriethoxysilane (APTES). Next, the immobilized naphthyridine diimine (NDI) ligand (NDI‐APTES@SBA‐15_250_) was synthesized via a condensation reaction between APTES@SBA‐15_250_ and the naphthyridine diketone. Finally, the heterogeneous material, Ni‐NDI‐APTES@SBA‐15_250_, was obtained by reacting NDI‐APTES@SBA‐15_250_ with Ni(COD)_2_ as a Ni(0) source, in which the redox‐active NDI sites promote the formation of a surface‐anchored bimetallic Ni species, mirroring the molecular analogue [44].

**SCHEME 2:**
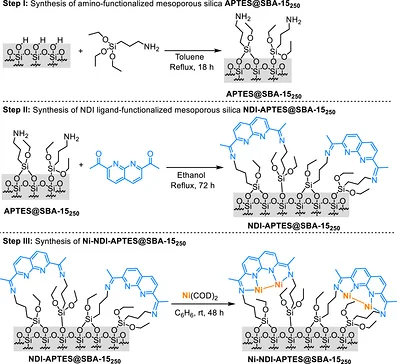
Outline for the synthesis of Ni‐NDI‐APTES@SBA‐15_250_ material.

For the synthesis of Ni‐NDI‐APTMS@SBA‐15_250_, an analogous procedure was followed, except that 3‐aminopropyltrimethoxysilane (APTMS) was used instead of APTES in the first step. The chemical and physical parameters of the silica support and its corresponding functionalized materials are presented in Table 1.

**TABLE 1:** Elemental analysis and textural parameters of the support material and functionalized SBA‐15_250_.

Material	Elemental content (wt %)				Physical parameters			
	C^a^	N^a^	Ni^b^	N/Ni molar ratio	*a*_BET_ (m^2^ g^−1^)	*V*_pore_ (cm^3^ g^−1^)	*d*_pore_ (nm)^c^	Ni/*a*_BET_ (mol/m^2^)
SBA‐15_250_	—	—	—	—	780	1.04	7.7	—
APTES@SBA‐15_250_	11.51	1.88	—	—	399	0.59	6.6	—
NDI‐APTES@SBA‐15_250_	17.20	2.78	—	—	380	0.49	5.8	—
Ni‐NDI‐APTES@SBA‐15_250_	18.11	2.61	2.03	5.4:1	333	0.43	5.6	1.04 x 10^−6^
APTMS@SBA‐15_250_	9.35	1.97	—	—	464	0.67	6.4	—
NDI‐APTMS@SBA‐15_250_	15.46	2.61	—	—	413	0.55	6.3	—
Ni‐NDI‐APTMS@SBA‐15_250_	17.06	2.51	1.98	5.3:1	357	0.47	6.2	9.45 x 10^−7^

^a^ Determined by elemental analysis.

^b^ Nickel content determined by ICP‐OES after digestion in *conc*. HNO_3_.

^c^ Maximum pore size distribution determined by the BJH method from the adsorption branch; SBA‐15 support was pretreated at 250°C for 4 h, and all functionalized materials were pretreated at ambient temperature for 2 h.

DRIFT‐IR spectra were recorded for mesoporous silica (SBA‐15_250_), the amino‐functionalized support (APTES@SBA‐15_250_), the corresponding NDI‐functionalized support (NDI‐APTES@SBA‐15_250_), and the Ni‐coordinated derivative (Ni‐NDI‐APTES@SBA‐15_250_) (Figure 1a). Since the APTMS‐based materials show analogous spectral features, their spectra are reported in Figure S3.

**FIGURE 1:**
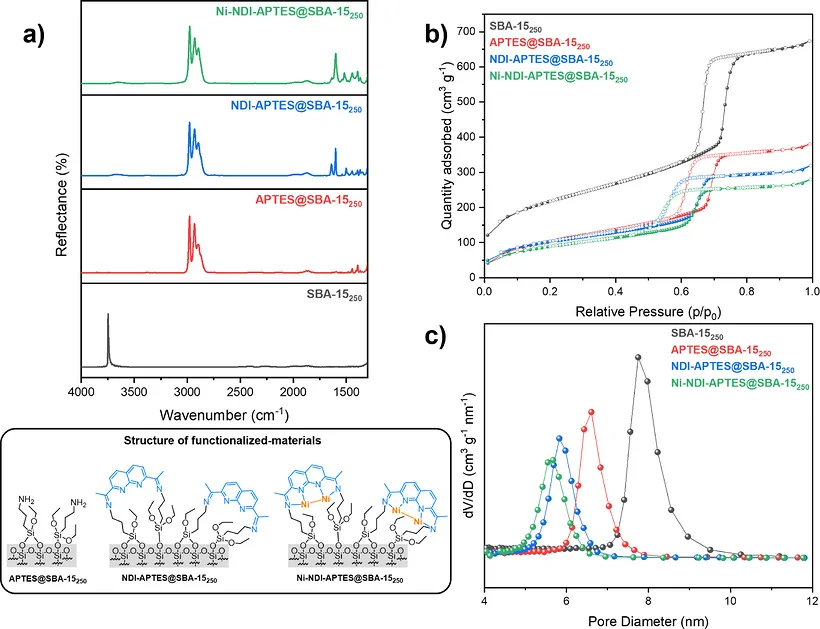
a) DRIFT‐IR spectra b) N_2_ physisorption isotherms and c) pore size distribution of SBA‐15_250_, APTES@SBA‐15_250_, NDI‐APTES@SBA‐15_250_, and Ni‐NDI‐APTES@SBA‐15_250_.

The amino‐functionalized material (APTES@SBA‐15_250_) featured the complete disappearance of the isolated silanol stretching band at 3746 cm^−1^ (*ν*_OH_) and the emergence of intense C–H stretching bands in the 2870–2980 cm^−1^ region, along with C–H bending vibrations at 1390–1500 cm^−1^, consistent with the presence of organic moieties and indicative of quantitative grafting of the amino groups [69, 70, 71]. For NDI‐APTES@SBA‐15_250_, new bands appeared at 1639 cm^−1^, assigned to C═N stretching, and at 1599 cm^−1^, attributed to C═C stretching of the phenyl ring backbone [33, 72, 73, 74, 75]. Moreover, the absence of the C═O band further confirmed the successful covalent immobilization of the NDI ligand on the amino‐functionalized support. Upon coordination of Ni to form Ni‐NDI‐APTES@SBA‐15_250_, the intensity of the band at 1639 cm^−1^ decreased markedly and shifted to a lower frequency [44, 75, 76]. This behavior suggests coordination of the Ni centers to the NDI ligand, in good agreement with analogous changes for the homogeneous dinuclear nickel complex (NDI)Ni_2_(C_6_H_6_) [44].

Elemental (CHN) microanalysis, combined with metal loading obtained from ICP‐OES, provides valuable information about the surface grafting process and the corresponding changes in the chemical composition of the functionalized materials at each functionalization step (Table 1). Both amino‐functionalized supports, APTES@SBA‐15_250_ and APTMS@SBA‐15_250_, displayed measurable C and N contents, confirming effective grafting of amino groups onto the silica surface. Subsequent modification with the NDI ligand to form NDI‐APTES@SBA‐15_250_ and NDI‐APTMS@SBA‐15_250_ led to further increases in C and N contents, indicating efficient grafting of the NDI ligand onto the amino‐functionalized supports. Upon Ni coordination, the Ni‐NDI‐APTES@SBA‐15_250_ and Ni‐NDI‐APTMS@SBA‐15_250_ materials showed Ni loadings of 2.03 wt% and 1.98 wt%, respectively, confirming incorporation of Ni centers within the functionalized frameworks. Nevertheless, the N/Ni molar ratio, calculated to be approximately 5:1 for both materials, exceeded the expected value of 2:1. This discrepancy is likely attributed to NDI ligand sites that remain completely uncoordinated within the mesoporous framework. Notably, a 1:1 Ni/ligand stoichiometry can be excluded, as XPS indicates predominantly a Ni_2_ coordination environment and the observed regioselectivities in catalysis closely match those of the corresponding molecular Ni_2_‐NDI complexes (*vide infra)*. The materials were synthesized using a 1:1 molar ratio of Ni(COD)_2_ relative to the total N content. In principle, formation of fully occupied Ni_2_ sites at each NDI unit would correspond to an N/Ni ratio of 2:1. However, under the applied conditions, complete coordination of all NDI ligands could not be achieved, likely due to diffusional and steric limitations within the mesoporous structure. Although DRIFT‐IR indicates that most aminopropyl groups were successfully converted during ligand immobilization, a minor fraction of residual unreacted amino groups (cf. Figure 2a) may still contribute to the overall N content determined by elemental analysis. As a result, only a fraction of the available ligand sites is coordinated to nickel, leading to the experimentally observed higher N/Ni ratio of approximately 5:1.

**FIGURE 2:**
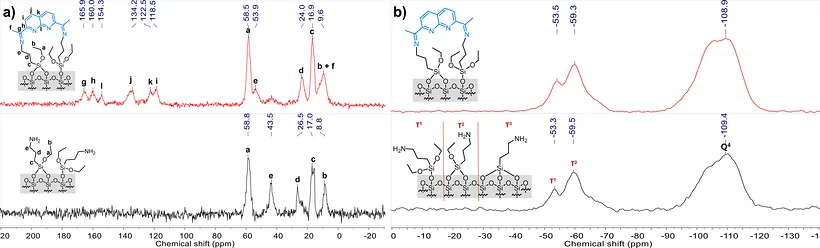
a) ^13^C CP/MAS NMR spectra of APTES@SBA‐15_250_ (black) and NDI‐APTES@SBA‐15_250_ (red), and b) ^29^Si CP/MAS NMR spectra of APTES@SBA‐15_250_ (black) and NDI‐APTES@SBA‐15_250_ (red).

The thermal properties of the functionalized SBA‐15_250_ materials were investigated by thermogravimetric analysis (TGA) and differential thermal analysis (DTA) to evaluate the successive surface modification steps and the stability of the immobilized organic and metal‐organic species (Figure S4). Pristine SBA‐15_250_ exhibited only minimal weight loss over the entire temperature range, mainly below 150°C, which is attributed to desorption of physically adsorbed water and condensation of surface silanol groups, confirming the high thermal stability of the silica framework. After amine functionalization, the APTES@SBA‐15_250_ material showed an increased weight loss compared with bare SBA‐15_250_, particularly between 250 and 600°C, corresponding to decomposition of grafted aminopropyl groups. The organic loading determined from the TGA analysis was approximately 18.4 wt%, confirming successful surface functionalization of SBA‐15_250_ with aminopropyl moieties. The NDI‐functionalized material exhibited a further increase in organic weight loss, corresponding to an organic content of approximately 22.2 wt%, indicating successful immobilization of the naphthyridine diimine (NDI) framework onto the amino‐functionalized surface. The broader decomposition profile observed for the NDI‐functionalized material suggests gradual thermal decomposition of the immobilized aromatic ligand framework over a wide temperature range. Upon nickel coordination, the Ni‐NDI‐APTES@SBA‐15_250_ material displayed the highest overall weight loss with an organic loading of approximately 28.1 wt%, together with a more pronounced thermal decomposition event around 350–450°C. This behavior is attributed to the decomposition of the coordinated Ni‐NDI surface species. Compared with the NDI‐functionalized material, the slightly lower thermal stability of the Ni‐NDI material suggests that nickel coordination modifies the thermal decomposition pathway of the immobilized ligand framework. In addition, nickel species may promote thermally induced ligand decomposition, leading to a more distinct thermal transition. The DTA profiles were consistent with the TGA results. The APTES@SBA‐15_250_ and Ni‐NDI‐APTES@SBA‐15_250_ materials exhibited noticeable thermal events associated with decomposition of grafted organic groups and coordinated metal‐ligand species. In contrast, the NDI‐functionalized material did not show a distinct DTA peak, indicating that decomposition of the immobilized aromatic ligand occurs gradually over a broad temperature range with less pronounced thermal transitions. Overall, the progressive increase in organic content from the amino‐functionalized to the NDI‐ and Ni‐NDI‐functionalized materials confirms successful stepwise surface modification of SBA‐15_250_. Combined with elemental analysis and ICP‐OES measurements, these results support successful immobilization of the NDI ligand framework and subsequent nickel coordination on the SBA‐15_250_ surface.

The BET surface areas (*a*_BET_), pore volume (*V*_pore_), and pore diameter (*d*_pore_) of the materials were determined from nitrogen physisorption isotherms, and the corresponding values are summarized in Table 1. The mesoporous silica support (SBA‐15_250_) and all immobilized materials exhibited type‐IV isotherms with hysteresis loops, characteristic of mesoporous structures (Figure 1b and Figure S5) [67, 69, 74, 77, 78].

The textural parameters, consisting of BET surface area, pore size, and pore volume, decreased gradually at each functionalization step from the SBA‐15_250_ support to the final materials, Ni‐NDI‐APTES@SBA‐15_250_ and Ni‐NDI‐APTMS@SBA‐15_250_. Moreover, the Ni‐NDI‐APTES@SBA‐15_250_ material displayed a lower surface area, pore size, and pore volume than the analogue Ni‐NDI‐APTMS@SBA‐15_250_, as expected, according to the bulkier amino‐functional group used in its synthesis. Consequently, when the Ni loading was determined per surface area, Ni‐NDI‐APTES@SBA‐15_250_ (1.04 x 10^−6^ mol/m^2^) showed a slightly higher Ni concentration per unit area than Ni‐NDI‐APTMS@SBA‐15_250_ (9.45 x 10^−7^ mol/m^2^).

To gain further insight into the structure of the amino‐functionalized silica and the covalently immobilized NDI ligand, the materials were analyzed by solid‐state MAS/NMR spectroscopy. The ^13^C CP/MAS NMR spectra of APTES@SBA‐15_250_ and NDI‐APTES@SBA‐15_250_ are shown in Figure 2a (spectra of the APTMS@SBA‐15_250_ and NDI‐APTMS@SBA‐15_250_ analogues are provided in Figure S6). The ^13^C CP/MAS NMR spectrum of APTES@SBA‐15_250_ confirms the successful grafting of the aminosilane onto the silica surface, as evidenced by five characteristic signals at 8.8, 17.0, 26.5, 43.5, and 58.8 ppm, corresponding to the carbon atoms of the APTES moiety [69, 77, 79]. The spectrum of NDI‐APTES@SBA‐15_250_ exhibits additional peaks in the 118–166 ppm region, corresponding to the aromatic carbons of the NDI ligand, in agreement with the ^13^C NMR spectrum of 1,1′‐(1,8‐Naphthyridine‐2,7‐diyl)diethanone (Figure S7). Moreover, the absence of the carbonyl (C═O) signal, characteristic of free ligand precursor in the spectra of the grafted material further supports the quantitative formation of imine groups on the silica surface. The ^29^Si CP/MAS NMR spectra of the various functionalized SBA‐15_250_ materials are shown in Figures 2b and S8. All spectra exhibit a resonance at approximately −109 ppm, corresponding to Q^4^ silicon sites [*Si*(OSi)_4_] [69, 80]. In addition, three resonances at around −51, −59, and −65 ppm are observed for all functionalized samples, which can be assigned to T^1^ [C‐*Si*(OR)_2_(OSi)], T^2^ [C‐*Si*(OR)(OSi)_2_] and T^3^ [C‐*Si*(OSi)_3_] species, respectively [69, 77, 81]. Deconvolution of these peaks for all functionalized samples indicates that T^2^ is the predominant species, followed by T^1^ and T^3^ (Figure S9 and Table S1). The presence of these signals confirms the covalent incorporation of organosilane moieties and reflects the formation of a well‐condensed siloxane framework with effective surface functionalization of SBA‐15_250_.

X‐ray photoelectron spectroscopy (XPS) was employed to gain deeper insight into the electronic structure of the heterogeneous material (Ni‐NDI‐APTES@SBA‐15_250_) and to compare directly with the molecular reference complex (NDI)Ni_2_(C_6_H_6_). The objective was to determine whether the Ni centers immobilized on the mesoporous silica support retain an electronic environment comparable to the homogeneous Ni(I)–Ni(I) complex. Notably, well‐characterized and reliable reference data for Ni(I) species in XPS are limited in the literature, because Ni(I) is relatively rare and unstable under normal conditions. Therefore, we synthesized and analyzed (NDI)Ni_2_(C_6_H_6_) to provide a direct experimental comparison [44]. Since Ni‐NDI‐APTES@SBA‐15_250_ and (NDI)Ni_2_(C_6_H_6_) are air‐sensitive, the sample preparation and handling were performed under an argon atmosphere to prevent oxidation. Both samples were mounted onto double‐sided conductive carbon tape inside a glovebox and subsequently transferred to the spectrometer using a custom‐built vacuum transfer device, thereby maintaining strictly inert conditions prior to analysis [82]. The Ni 2p core‐level spectra of Ni‐NDI‐APTES@SBA‐15_250_ and (NDI)Ni_2_(C_6_H_6_) exhibit an identical Ni 2p_3/2_ binding energy maximum at 858.4 eV, along with similar spin‐orbit splitting values of 17.8–17.9 eV (Figure S10). The close agreement in both peak position and splitting strongly suggests that the Ni centers in the heterogeneous material share a similar oxidation state and electronic configuration with the molecular complex. Overall, these spectroscopic similarities provide compelling evidence that the grafting procedure preserves the electronic identity of the dinuclear Ni centers. Therefore, the heterogeneous material maintains Ni species that are electronically comparable to the (NDI)Ni_2_(C_6_H_6_) complex. These results support the successful translation of the molecular Ni_2_ motif into a surface‐anchored environment.

### 2.2 Catalyst Performance Studies

The catalytic performance of Ni‐NDI‐APTES@SBA‐15_250_ was further evaluated in the cyclotrimerization of several terminal alkynes bearing various electronic properties (Table 2) to assess whether the immobilized Ni centers operate as a cooperative dinuclear unit with a short Ni–Ni distance. This specific transformation is known to exhibit a characteristic regioselectivity when mediated by dinuclear nickel species, which has been attributed to cooperative interactions of two proximal Ni centers with the reacting alkynes [46, 83]. The reaction conditions were optimized for each substrate by varying the solvent, catalyst loading (mol% Ni), reaction temperature, and reaction time. Furthermore, all substrates were also evaluated in the absence of the catalyst as control experiments, and no conversion was observed (Table 2, entries 1, 8, and 14). Phenylacetylene was used as a benchmark terminal alkyne to assess the catalytic performance of Ni‐NDI‐APTES@SBA‐15_250_ and Ni‐NDI‐APTMS@SBA‐15_250_ as heterogeneous catalysts. APTES@SBA‐15_250_ and NDI‐APTES@SBA‐15_250_ were evaluated as control materials to determine whether free amine groups or non‐nickel‐coordinated frameworks could promote cyclotrimerization. In both cases, no substrate conversion was observed, indicating that the amine‐functionalized and ligand‐immobilized materials alone are catalytically inactive in the absence of coordinated nickel species (Table 2, entries 2 and 3, respectively).

**TABLE 2:** Reactivity of Ni‐NDI‐APTES@SBA‐15_250_ in cyclotrimerization with the terminal alkynes phenylacetylene, ethyl propiolate, and methyl propargyl ether.^a^

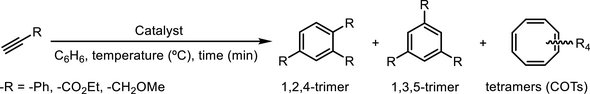							
Entry	Substrate (‐R)	Catalyst	Amount of catalyst (mol% Ni)	Conversion (%)**^b^**	Selectivity (%)**^c^**		
					1,2,4	1,3,5	COTs
1	‐Ph (**I**)	No catalyst	—	<1	*n.d*.	*n.d*.	*n.d*.
2	‐Ph (**I**)	APTES@SBA‐15_250_	40 mg	<1	*n.d*.	*n.d*.	*n.d*.
3	‐Ph (**I**)	NDI‐APTES@SBA‐15_250_	40 mg	<1	*n.d*.	*n.d*.	*n.d*.
4	‐Ph (**I**)	Ni‐NDI‐APTES@SBA‐15_250_	2.5	84	89	11	—
5	‐Ph (**I**)	Ni‐NDI‐APTES@SBA‐15_250_	3.75	98	87	13	—
6	‐Ph (**I**)	Ni‐NDI‐APTES@SBA‐15_250_	5	>99	80	20	—
7	‐Ph (**I**)	Ni‐NDI‐APTMS@SBA‐15_250_	3.75	95	76	24	—
8	‐CO_2_Et (**V**)	No catalyst	—	<1	*n.d*.	*n.d*.	*n.d*.
9	‐CO_2_Et (**V**)	Ni(COD)_2_	1	94	23	14	56
10	‐CO_2_Et (**V**)	Ni‐NDI‐APTES@SBA‐15_250_	1	63	72	28	—
11^d^	‐CO_2_Et (**V**)	Ni‐NDI‐APTES@SBA‐15_250_	1	72	76	24	—
12	‐CO_2_Et (**V**)	Ni‐NDI‐APTES@SBA‐15_250_	2	97	79	21	—
13	‐CO_2_Et (**V**)	Ni‐NDI‐APTMS@SBA‐15_250_	2	96	75	25	—
14	‐CH_2_OMe (**VI**)	No catalyst	—	<1	*n.d*.	*n.d*.	*n.d*.
15	‐CH_2_OMe (**VI**)	Ni‐NDI‐APTES@SBA‐15_250_	1	55	74	26	—
16	‐CH_2_OMe (**VI**)	Ni‐NDI‐APTES@SBA‐15_250_	2	64	74	26	—
17^e^	‐CH_2_OMe (**VI**)	Ni‐NDI‐APTES@SBA‐15_250_	2	98	75	25	—
18^e^	‐CH_2_OMe (**VI**)	Ni‐NDI‐APTMS@SBA‐15_250_	2	92	74	26	—

^a^ Reaction conditions for phenylacetylene 0.4 mmol as a substrate, mesitylene 0.4 mmol as a standard, C_6_H_6_ 0.5 mL as a solvent, and catalyst 2.5‐5 mol % Ni at 60°C for 40 min. Reaction conditions for ethyl propiolate and methyl propargyl ether 0.4 mmol as a substrate, mesitylene 0.4 mmol as a standard, and catalyst 1–2 mol % Ni at ambient temperature for 11 min.

^b^ Conversion was determined by ^1^H NMR using mesitylene as an internal standard.

^c^ Selectivity was determined by GC‐MS.

^d^ The reaction was carried out at 60°C.

^e^ The reaction was carried out for 60 min.

The effect of solvent was subsequently investigated using benzene, toluene, THF, and acetonitrile (Table S2). Among the solvents examined, benzene afforded the highest catalytic performance, followed by toluene, while THF resulted in lower activity. A significant decrease in conversion was observed in acetonitrile. The superior performance in benzene is likely associated with its weak coordinating nature, which minimizes competitive interactions with the nickel active sites and facilitates efficient alkyne coordination. In contrast, the stronger coordinating ability of acetonitrile likely inhibits substrate binding to the nickel centers, leading to reduced catalytic activity. Accordingly, benzene was selected as the optimal solvent for further reaction optimization. The substrate conversion significantly depends on the catalyst loading. Increasing the catalyst amount from 2.5 to 5 mol% Ni enhanced the conversion from 84% to quantitative conversion, accompanied by a slight decrease in the ratio of 1,2,4‐trimer to 1,3,5‐trimer products. Notably, under all conditions tested, the reaction showed excellent selectivity, affording only trimeric products without formation of cyclooctatetraenes (COTs) (Table 2, entries 4–6). Additionally, Ni‐NDI‐APTMS@SBA‐15_250_, which possesses a less sterically hindered environment and exhibits higher surface area, larger pore size and greater pore volume than Ni‐NDI‐APTES@SBA‐15_250_ showed comparable catalytic activity to Ni‐NDI‐APTES@SBA‐15_250_ under identical reaction conditions (3.75 mol% Ni, 60 °C, 40 min), affording 95% conversion along with a slightly lower ratio of 1,2,4‐ to 1,3,5‐trimer (Table 2, entry 7). Based on the results found with phenylacetylene, Ni‐NDI‐APTES@SBA‐15_250_ was selected for further optimization and investigation using other terminal alkynes, including ethyl propiolate and methyl propargyl ether. Moreover, the catalytic performance of Ni‐NDI‐APTES@SBA‐15_250_ was also compared with that of the analogous material, Ni‐NDI‐APTMS@SBA‐15_250_.

For the electron‐deficient ethyl propiolate, the performance of commercial Ni(COD)_2_ as a homogeneous Ni(0) catalyst was investigated for comparison with Ni‐NDI‐APTES@SBA‐15_250_. Ni(COD)_2_ showed high conversion (94%) but very low selectivity toward trimeric products, predominantly forming cyclooctatetraenes (COTs) under the condition of 1 mol% Ni at ambient temperature for 11 min (Table 2, entry 9), consistent with a previous report [46]. In comparison, when Ni‐NDI‐APTES@SBA‐15_250_ was used under identical conditions, a moderate conversion (63%) was obtained with remarkable selectivity, affording only trimeric species in an approximate 3:1 ratio of 1,2,4‐ to 1,3,5‐trimers (Table 2, entry 10). To enhance the catalytic activity, the reaction conditions were further optimized. When the reaction temperature was increased to 60°C, a slightly higher conversion (72%) was achieved, with no significant change in selectivity (Table 2, entry 11). Moreover, increasing the catalyst loading to 2 mol% Ni resulted in nearly quantitative conversion (97%), while maintaining a similar ratio of the trimeric products (Table 2, entry 12). Finally, the analogous material, Ni‐NDI‐APTMS@SBA‐15_250,_ was evaluated under optimized conditions and exhibited comparable catalytic performance, achieving 96% conversion with a slightly lower 1,2,4 to 1,3,5‐trimeric ratio relative to Ni‐NDI‐APTES@SBA‐15_250_ (Table 2, entry 13).

For the electron‐rich methyl propargyl ether, the conversion was more strongly influenced by the reaction conditions. Only 55% conversion was found after 11 min using 1 mol% Ni of Ni‐NDI‐APTES@SBA‐15_250_ at ambient temperature. Increasing the catalyst loading to 2 mol% Ni improved the conversion (64% after 11 min), and nearly complete conversion (98%) was obtained after 60 min. Notably, across all reaction conditions, increased catalyst loading and extended reaction time consistently maintained an approximate 3:1 ratio of 1,2,4‐ to 1,3,5‐trimeric products (Table 2, entries 15–17). Furthermore, Ni‐NDI‐APTMS@SBA‐15_250_ was evaluated under the optimized conditions (2 mol% Ni, 60 min) and showed slightly lower catalytic activity, achieving 92% conversion while maintaining a trimeric product distribution similar to that observed for Ni‐NDI‐APTES@SBA‐15_250_.

Based on the catalytic comparison between Ni‐NDI‐APTES@SBA‐15_250_ and Ni‐NDI‐APTMS@SBA‐15_250_, only minor differences in activity and product distribution were observed, indicating that variations in the local environment and steric properties around the nickel centers do not significantly affect the catalytic performance. Instead, the results suggest that the surface‐confined dinuclear active sites play the dominant role in promoting cyclotrimerization. Importantly, both catalysts exhibited high catalytic activity and selectivity toward trimeric products with predominant formation of the 1,2,4‐isomer. The observed catalytic behavior is consistent with the previous report on the homogeneous dinuclear nickel naphthyridine diimine complex [46], further supporting the presence of surface‐bound Ni_2_NDI active species, in agreement with the XPS analysis.

To differentiate mononuclear from dinuclear reactivity and assess whether Ni–Ni cooperativity operates in the supported system, Ni‐NDI‐APTES@SBA‐15_250_ was compared with homogeneous mononuclear and dinuclear nickel complexes reported by Uyeda and Groysman (Figure 3a, representative structures and Figure 4, catalytic performance comparison) [46, 83].

**FIGURE 3:**
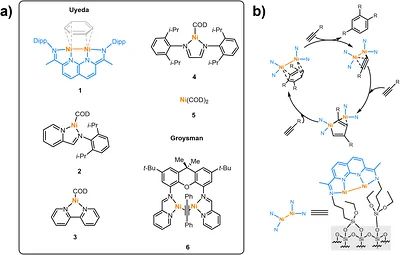
(a) Mononuclear Ni complexes and dinuclear Ni complexes reported by Uyeda and Groysman [46, 83] and (b) Proposed reaction mechanism for Ni‐NDI‐APTES@SBA‐15_250_ in the cyclotrimerization reaction [46, 83, 84].

**FIGURE 4:**
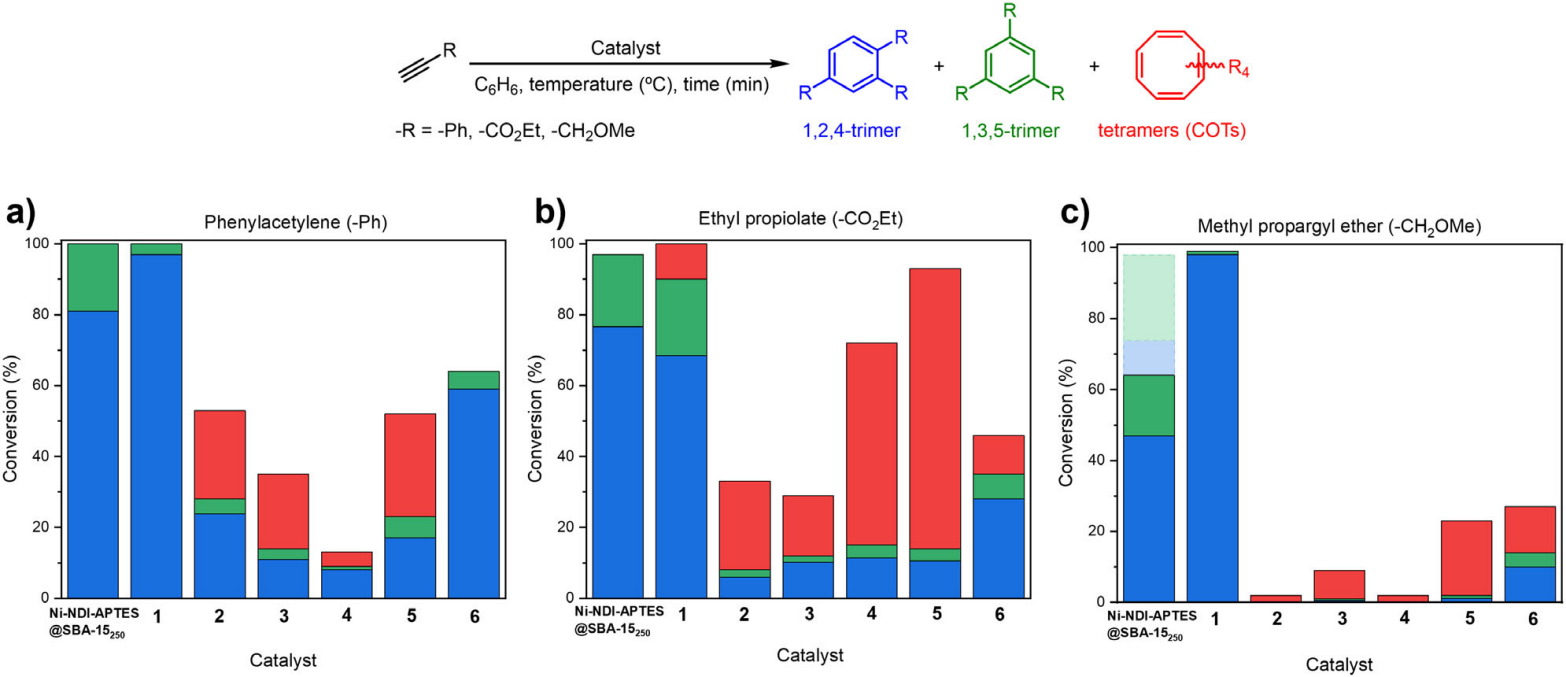
Catalytic comparison of Ni‐NDI‐APTES@SBA‐15_250_ with previously reported mononuclear‐ and dinuclear Ni complexes. Synthetic procedures and catalytic data for the reported complexes **1–6** were obtained from literature [46, 83]. Reaction conditions: (a) phenylacetylene: 5 mol% Ni, 60°C, 40 min, and C_6_H_6_ as solvent. *Note*: for complexes **1** and **6**, 5 mol% catalyst corresponds to 10 mol% Ni. (b) ethyl propiolate: 2 mol% Ni, room temperature, 11 min, and C_6_H_6_ as solvent. (c) methyl propargyl ether: 2 mol% Ni, room temperature, 11 min (60 min presented in lighter colors), and C_6_H_6_ as solvent. The total heights of the bars represent the overall conversions of the starting materials. The products corresponding to the 1,2,4‐trimer (solid blue), 1,3,5‐trimer (solid green) and other tetrameric products (solid red) are shown. The results are provided in tabular form in Table S3.

In the cyclotrimerization of phenylacetylene (Figure 4a), Ni‐NDI‐APTES@SBA‐15_250_ showed catalytic performance comparable to the homogeneous (NDI)Ni_2_(C_6_H_6_) (**1**), yielding quantitative conversion with exclusive formation of trimeric products and a strong preference for the 1,2,4‐isomer using 5 mol% catalyst, 60°C, 40 min. Notably, a lower amount of active nickel species was employed in the heterogeneous system: Ni‐NDI‐APTES@SBA‐15_250_ contains 5 mol% Ni, whereas the homogeneous system utilized 5 mol% of **1**, corresponding to 10 mol% Ni as the active metal species [46]. This comparison highlights the higher efficiency of the heterogeneous catalyst relative to the homogeneous analogue. Consistent with this observation, Ni‐NDI‐APTES@SBA‐15_250_ exhibited a higher turnover number (TON) than complex **1** under identical conditions (Table S3), demonstrating that comparable catalytic performance can be achieved with substantially lower nickel loading. These results suggest that the immobilized dinuclear nickel active sites remain highly accessible and catalytically competent within the SBA‐15_250_ framework. The dinuclear Ni xanthene‐bridged‐bis(iminopyridine) complex (**6**) displayed lower catalytic activity, although it maintained high selectivity toward the 1,2,4‐trimer due to its cooperative dinuclear Ni sites. In the cyclotrimerization of ethyl propiolate (Figure 4b), Ni‐NDI‐APTES@SBA‐15_250_ showed slightly lower activity than complex **1** under identical conditions (2 mol% Ni, ambient temperature, 11 min). Nevertheless, Ni‐NDI‐APTES@SBA‐15_250_ afforded only trimeric products, whereas complex **1** generated a mixture of trimers and minor amounts of tetrameric byproducts (COTs). Compared with complex **6**, Ni‐NDI‐APTES@SBA‐15_250_ achieved both higher conversion and complete selectivity toward trimer formation, while complex **6** also produced tetramers (COTs). These results indicate that, although Ni‐NDI‐APTES@SBA‐15_250_ shows slightly reduced activity relative to complex **1** for this substrate, it provides better product selectivity. Therefore, when TON was determined based on the desired 1,2,4‐trimeric product, Ni‐NDI‐APTES@SBA‐15_250_ exhibited a slightly higher TON than the homogeneous analogue due to its complete suppression of tetramer formation. In the cyclotrimerization of methyl propargyl ether (Figure 4c), Ni‐NDI‐APTES@SBA‐15_250_ exhibited lower initial catalytic activity than complex **1** under identical reaction conditions (2 mol% Ni, ambient temperature, 11 min). This reduced activity may be attributed to slower dispersion of the heterogeneous catalyst in the reaction medium or to an induction period associated with activation of the surface‐bound species. Consistent with this observation, Ni‐NDI‐APTES@SBA‐15_250_ exhibited a lower TON than complex **1** under this condition. Upon extending the reaction time to 60 min, however, nearly quantitative conversion was achieved (Figure 4c, light color). Both Ni‐NDI‐APTES@SBA‐15_250_ and complex **1** showed exclusive selectivity toward trimeric products, further emphasizing the crucial role of dinuclear Ni sites in controlling product distribution. In contrast, the dinuclear complex **6** exhibited lower catalytic activity along with reduced selectivity for this substrate. Overall, both Ni‐NDI‐APTES@SBA‐15_250_ and complex **1**, which feature dinuclear Ni–Ni active sites, exhibited excellent selectivity for trimeric products, particularly favoring the 1,2,4‐isomer [46]. Interestingly, the heterogeneous Ni‐NDI‐APTES@SBA‐15_250_ catalyst produced only trimeric products across all substrates, likely due to surface confinement and the structural rigidity of the grafted Ni_2_‐NDI framework, which favors selective cyclotrimerization pathways. Furthermore, compared with related homogeneous mononuclear Ni catalysts (**2**‐**5**), Ni‐NDI‐APTES@SBA‐15_250_ showed markedly higher activity and selective formation of the trimeric 1,2,4‐product, highlighting the essential role of Ni–Ni cooperativity in driving efficient and selective cyclotrimerization.

These results from the catalytic comparison highlight the important role of the Ni–Ni active sites, which further support the formation of dinuclear nickel complexes in Ni‐NDI‐APTES@SBA‐15_250_. These sites promote high activity and selectivity toward cyclotrimerized 1,2,4‐products by stabilizing key metallacyclopentadiene intermediates, as indicated in the proposed reaction mechanism for the Ni‐NDI‐APTES@SBA‐15_250_ heterogeneous catalyst (Figure 3b).

To evaluate the effect of catalyst immobilization on catalytic performance, the substrate scope of Ni‐NDI‐APTES@SBA‐15_250_ was compared with that of the corresponding homogeneous dinuclear nickel catalyst (**1**) (Figure 5). Overall, Ni‐NDI‐APTES@SBA‐15_250_ exhibited excellent catalytic performance across a diverse range of terminal alkynes, demonstrating comparable or superior activity while maintaining outstanding selectivity. For aromatic alkynes **I‐III**, Ni‐NDI‐APTES@SBA‐15_250_ consistently afforded higher conversions than the homogeneous catalyst with exclusive formation of cyclotrimerization products, demonstrating that immobilization preserves, and in these cases enhances, catalytic performance. The only exception was the sterically demanding substrate **IV**, for which Ni‐NDI‐APTES@SBA‐15_250_ was inactive, suggesting that increased steric congestion limits substrate accessibility to the functionalized catalytic sites. In contrast, substrate **V** highlights a clear advantage of the heterogeneous catalyst. Although both catalytic systems afforded comparable conversions, the homogeneous catalyst (**1**) produced both cyclotrimerization and cyclooctatetraene (COT) products, whereas Ni‐NDI‐APTES@SBA‐15_250_ exclusively yielded the cyclotrimerization product, demonstrating that the heterogeneous catalyst effectively suppresses the competing tetramerization pathway while maintaining catalytic activity. For the more flexible substrates **VI**‐**VIII**, the homogeneous dinuclear nickel catalyst (**1**) afforded somewhat higher conversions; however, Ni‐NDI‐APTES@SBA‐15_250_ still delivered good to excellent conversions (54%–97%) with excellent selectivity, indicating that catalyst immobilization primarily influences the apparent reaction rate rather than the intrinsic cyclotrimerization capability. Most notably, the diyne substrates **IX** and **X** revealed a distinct chemoselectivity that is unique to the heterogeneous catalyst. Whereas the homogeneous Ni_2_(NDI) catalyst (**1**) predominantly promoted sequential cyclotrimerization of both terminal alkynes to afford the doubly cyclized products **IXa** and **Xa**, Ni‐NDI‐APTES@SBA‐15_250_ selectively produced the corresponding monocyclized products **IXb** and **Xb**. Notably, products analogous to **IXb** and **Xb** have previously been reported primarily as side products in transition‐metal‐catalyzed (group 8 and 9) diyne cyclotrimerization reactions, whereas the corresponding double cyclized products are generally favored under optimized homogeneous conditions [85, 86, 87, 88, 89, 90]. The selective formation of **IXb** and **Xb** by Ni‐NDI‐APTES@SBA‐15_250_, therefore, represents a distinct chemoselectivity induced by catalyst functionalization. Overall, these results demonstrate that immobilization of the dinuclear Ni catalyst on SBA‐15 provides an effective strategy for tuning catalytic activity, enhancing product selectivity, suppressing undesired side reactions, and achieving chemoselective transformations that are not accessible with the corresponding homogeneous molecular catalyst.

**FIGURE 5:**
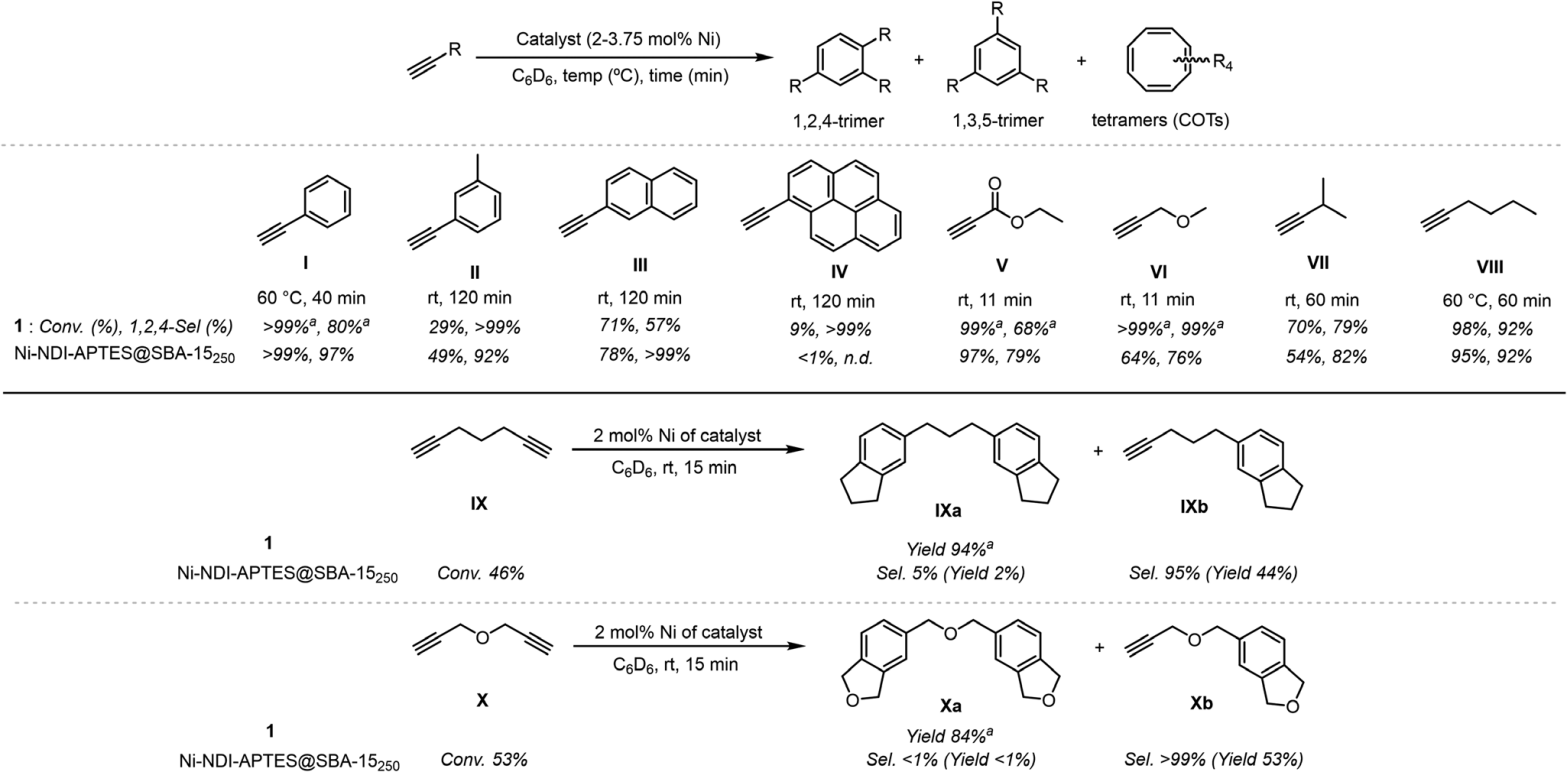
Comparison of the substrate scope and catalytic performance of heterogeneous Ni‐NDI‐APTES@SBA‐15_250_ and previously reported Ni_2_(NDI) (**1**) catalysts. Reaction conditions: 3.75 mol% Ni loading for **VII** and **VIII** substrates and 2 mol% Ni for all other substrates. *a* previously reported in [46]. The results are summarized in Table S4.

### 2.3 Catalyst Recyclability

Ni‐NDI‐APTES@SBA‐15_250_ was further evaluated to assess its stability under operating conditions and to highlight the robustness of the heterogeneous system. Rather than isolating the catalyst between cycles, the initial reusability assessment was performed by the addition of fresh substrate during the reaction. A new portion of substrate was introduced every 20 min without removing or recovering the catalyst, ensuring that no material loss occurred during handling. The catalytic performance over the four consecutive catalytic cycles is shown in Figure 6. The catalytic conversion decreased slightly from 76% in the first cycle to 72% in the second, followed by a more pronounced drop to 61% in the third run and finally 49% in the fourth cycle. Notably, the selectivity toward the trimeric products remained unchanged at 100% throughout the recycling experiments.

**FIGURE 6:**
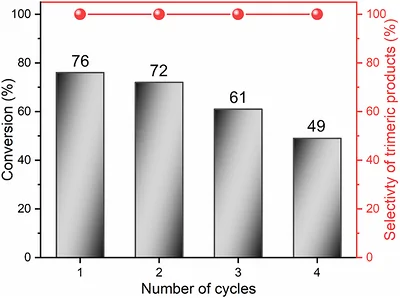
Recycling performance of Ni‐NDI‐APTES@SBA‐15_250_ over four consecutive catalytic cycles at every 20 min.

To further verify the heterogeneous nature of the catalyst and exclude the possibility of metal leaching, a hot filtration test was performed by removing the catalyst after 20 min and allowing the reaction mixture to continue to 40 min (Figure S11). The conversion remained unchanged at 76%, indicating negligible leaching of active Ni species under the reaction conditions and confirming that catalysis occurs only at the solid surface. Moreover, the low‐angle PXRD patterns of the fresh and reused catalysts exhibited characteristic reflections corresponding to the 2D hexagonal mesostructure (Figure S12). These results indicate that the structural ordering and symmetry of the material were well preserved even after four catalytic cycles, demonstrating that the mesoporous framework remained intact during the reuse experiments. Furthermore, the reused material was investigated by N_2_ physisorption (Figure S13) and the results are provided in Table S7. The data indicate slight reductions in surface area, pore volume, and pore diameter. These changes suggest the partial trapping of organic compounds within the pores during catalytic cycles, which is consistent with the DRIFT spectra of the reused catalyst (Figure S14) showing distinct signals between 3028–3086 cm^−1^, corresponding to C–H stretching vibrations of aromatic compounds, indicating the presence of adsorbed aromatic products on the surface and inside the pores. This adsorption likely contributes to the observed decreases in textural properties. The SEM and TEM analyses of the reused catalyst showed no significant changes compared to the fresh catalyst, indicating that the support remains structurally stable (Figures 7, S15, and S16).

**FIGURE 7:**
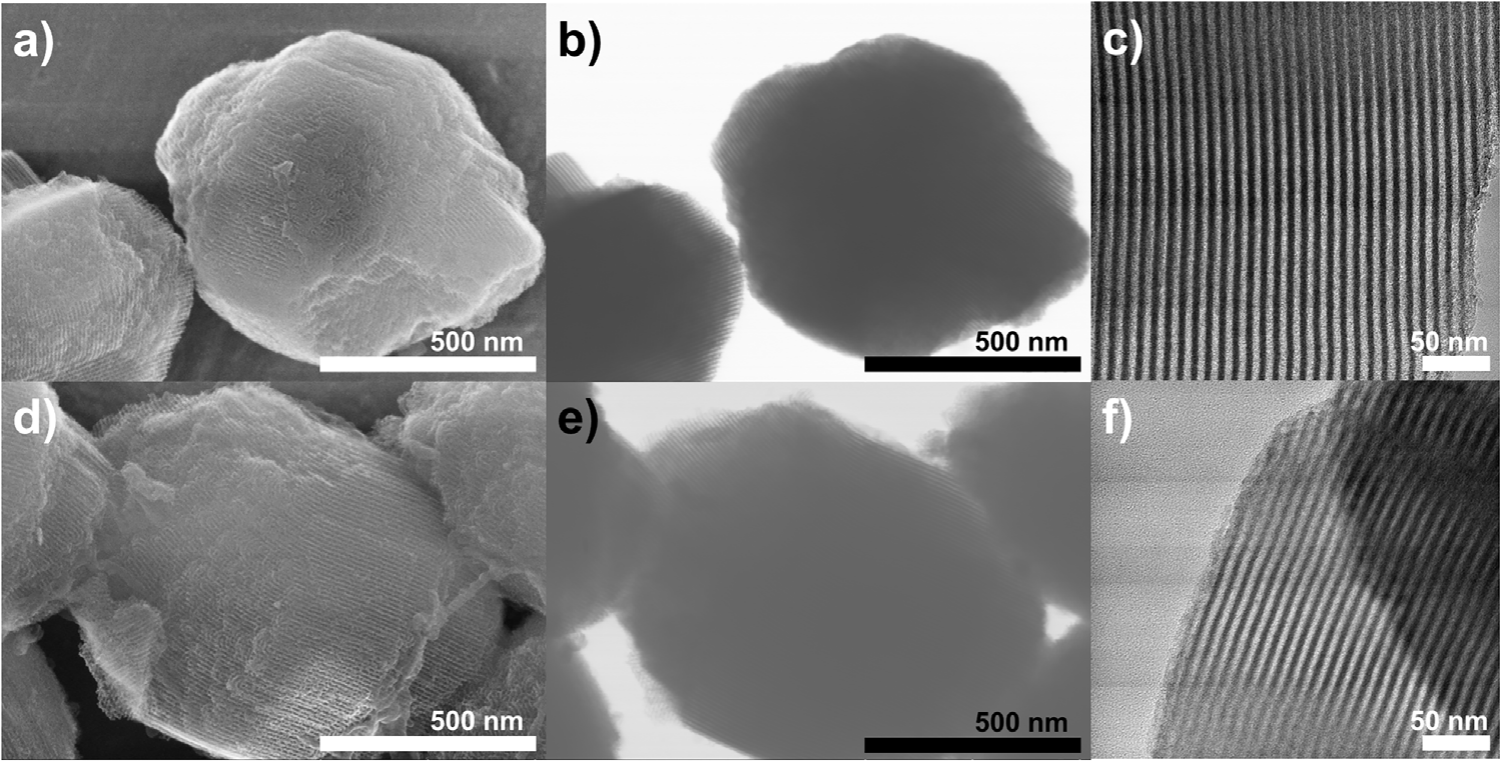
Representative SEM/TEM and HRTEM of (a–c) Ni‐NDI‐APTES@SBA‐15_250_ (Fresh) and (d–f) Ni‐NDI‐APTES@SBA‐15_250_ (Reused).

Additionally, HRTEM images showed clearly preserved mesoporous channels of SBA‐15 in both the fresh and reused materials (Figures 7, S17, and S18), further demonstrating the high structural stability of the material. STEM‐EDX mapping confirmed homogeneous nickel distribution in both fresh and reused catalysts, indicating that the nickel species remain well dispersed on the support after catalysis (Figures S19 and S20). Therefore, the observed decrease in catalytic performance is most likely due to the loss of finely powdered immobilized silica catalyst during the catalytic cycle process rather than metal leaching or support instability.

## 3 Conclusion

In summary, we have demonstrated that the cooperative reactivity characteristic of NDI‐Ni_2_ complexes can be successfully transferred to a heterogeneous material (Ni‐NDI‐APTES@SBA‐15_250_) through a systematic immobilization strategy. The formation of functionalized heterogeneous materials at each stage was thoroughly characterized. Furthermore, Ni‐NDI‐APTES@SBA‐15_250_ exhibits excellent catalytic performance in terminal alkyne cyclotrimerization, affording high activity and outstanding selectivity toward 1,2,4‐trisubstituted benzene. Importantly, substrate‐scope investigations revealed that the functionalized catalyst not only preserves the characteristic reactivity of the molecular dinuclear Ni catalyst but also introduces substrate‐dependent catalytic behavior. In particular, the immobilized catalyst exhibits distinct chemoselectivity for diyne substrates, selectively affording mono‐cyclotrimerization products that differ from those obtained with the homogeneous analogue, highlighting the influence of the confined catalytic environment on the reaction pathway. Furthermore, Ni‐NDI‐APTES@SBA‐15_250_ retains its performance over multiple cycles, exhibits negligible nickel leaching, maintains 100% selectivity, and experiences only a moderate decrease in conversion upon reuse. Overall, this study presents a strategy for transferring molecular bimetallic design principles to solid supports, resulting in a robust, reusable heterogeneous catalyst that retains the key cooperative features and maintains high catalytic efficiency. Current efforts in our laboratory are focused on broadening the scope of this heterogeneous catalyst to additional transformations.

## Author Contributions

**Jitpisut Poolwong**: conceptualization, writing – original draft, investigation, methodology, validation, visualization, writing – review and editing, formal analysis. **Adrian Jenner**: investigation, writing – review and editing, writing – original draft. **Eric Juriatti**: investigation, writing – original draft, writing – review and editing. **Mustafa Turan**: investigation. **Manfred Manßen**: conceptualization, funding acquisition, writing – original draft, validation, visualization, writing – review and editing, project administration, data curation, supervision, resources.

## Conflicts of Interest

The authors declare no conflicts of interest.

## Supporting information




**Supporting File 1**: anie74247‐sup‐0001‐SuppMat.docx.

## Data Availability

The data that support the findings of this study are available in the supplementary material of this article
