# Supplementary material for: Defined Dinuclear Nickel Sites on Mesoporous Silica Translate Molecular Surface Mimics to Heterogeneous Catalysis
Source: Angew Chem Int Ed Engl. 2026 Aug 23;65(42):e5073615. doi: 10.1002/anie.5073615 (PMC13653570; doi:10.1002/anie.5073615)
Supplement: Supplementary file 1 [file ANIE-65-e5073615-s001.docx]

**Supporting Information**

**Defined Dinuclear Nickel Sites on Mesoporous Silica Translate Molecular Surface Mimics to Heterogeneous Catalysis**

*Jitpisut Poolwong,^a^ Adrian Jenner,^a^ Eric Juriatti,^b^ Mustafa Turan^c^ and Manfred Manßen ^a^**

*^a^*Institut für Anorganische Chemie, Eberhard Karls Universität Tübingen, 72076 Tübingen, Germany, E-mail: manfred.manssen@uni-tuebingen.de

*^b^*Institut für Physikalische und Theoretische Chemie, Eberhard Karls Universität Tübingen, 72076 Tübingen, Germany.

*^c^*Institut für Technische Chemie, Universität Stuttgart, 70569 Stuttgart, Germany.

**Table of Contents**

S1. Experimental Procedures………………………………………………………….…………..3

S2. Materials and Compounds Characterization…………………………………..………….……9

S3. Solid-State NMR Spectroscopy of Grafted Materials………………………………………..13

S4. XPS (X-ray Photoelectron) Spectroscopy……………………………………………………18

S5. Procedure for the Catalytic Reactions………………………………………………………..19

S6. Solvent Effect in Cyclotrimerization………………………………………………………...20

S7. Catalytic Comparison to Previous Reports…………………………………………………..21

S8. Reusability Study…...………………………………………………………………………..25

S9. NMR Spectra of Crude Mixtures from Representative Catalytic Reactions...……...……….34

S10. GC Chromatograms of Crude Mixtures from Representative Catalytic Reactions...………38

**S1. Experimental Procedures**

General Procedures

All manipulations were performed under an argon atmosphere using glovebox (MBraun MB MB150B-G-II; <1 ppm O_2_, <1 ppm H_2_O) and standard Schlenk techniques in oven-dried glassware. Solvents were purified by Grubbs-type columns (Mbraun SPS system) and stored in the glovebox. Deuterated solvent C_6_D_6_ was dried over Na/K alloy and filtered prior to use, whereas CDCl_3_ was used as received. All other reagents and starting materials were purchased from commercial vendors and used without further purification. Liquid reagents were degassed and stored over activated molecular sieves prior to use. GC-MS measurements were performed on an Agilent 8890 GC system equipped with a 5977B mass selective detector (MSD). Solution NMR spectra were recorded on a Bruker AVII+400 (^1^H: 400.13 MHz, ^13^C: 125.76 MHz). Solid-state NMR spectra were obtained on a Bruker ASX 300 spectrometer (^1^H 300.13 MHz, ^13^C 75.47 MHz, and ^29^Si 59.62 MHz) using 4 mm ZrO_2_ rotors. DRIFT-IR spectra were collected on a Bruker INVENIO R (ṽ = 4000-400 cm^-1^) using a DRIFT cell with dry KBr/sample mixtures and KBr windows. Elemental analyses (CHNS) were performed on an Elementar Vario Micro Cube analyzer. Metal contents were determined by ICP-OES (Thermo Scientific iCAP 7000). N_2_ physisorption isotherms were measured on a Micromeritics ASAP 2020 analyzer. Samples were degassed at 250 °C for 4 h (pure silica material) or at ambient temperature for 2 h (grafted materials) to prevent degradation of surface-bound organic/organometallic species. Low-angle powder X-ray diffraction (PXRD) patterns were collected on a Bruker D8 Advance diffractometer using monochromatic Cu K*α* radiation (λ = 1.5406 Å). Morphological analyses were performed by scanning and transmission electron microscopy (SEM/TEM) on a Hitachi SU8030 instrument.

XPS analysis was performed under ultra-high vacuum (UHV) conditions (8 $\text{×}$ 10^-10^ mbar) using an XR50 Al K*α* standard source (*hν*= 1486.6 eV) equipped with a PHOIBOS 100 hemispherical analyser (SPEC GmbH, Berlin, Germany). The binding energy was calibrated to the signal positions of Au 4f_7/2_ (84.00 eV), Ag 3d_5/2_ (368.21 eV) and Cu 2p_3/2_ (932.63 eV). To avoid exposure to air and prevent oxidation, the samples were mounted on a double-sided conductive carbon tape under an argon atmosphere and then transferred into the spectrometer using a custom-built vacuum transfer device.^1^

**Materials Synthesis and Preparation**

Scheme S1. Synthetic procedures to produce 1,1′-(1,8-Naphthyridine-2,7-diyl)diethanone (**C**).

2-Ethyl-1,8-naphthyridine (**A**). According to a reported procedure, with minor modifications.^2^ 2-aminonicotinaldehyde (12.5 g, 0.1 mol) and ethanol (90 mL) were charged into a Schlenk flask. Pyrrolidine (8.66 g, 0.12 mol) and H_2_SO_4_ (0.3 mL) were then added to the reaction mixture, which was stirred and heated to 65 °C. Subsequently, 2-butanone (10.55 g, 0.15 mol) was added dropwise over 90 min. The reaction mixture was stirred for an additional 1 h, affording the yellow crude product. Then the crude product was further purified by column chromatography (SiO_2_, 100% EtOAc) to yield 2-Ethyl-1,8-naphthyridine (**A**) as a yellow-orange product (45% yield). ^1^H NMR (400.13 MHz, Chloroform-d): *δ* = 9.05 (dd, *J* = 6.2 Hz, 2.2 Hz, 1H), 8.12 (dd, *J* = 8.0 Hz, 2.0 Hz, 1H), 8.07 (d, *J* = 8.3 Hz, 1H), 7.47-7.27 (m, 2H), 3.06 (q, *J* = 7.6 Hz, 1H), 1.42 (t, *J* = 7.6 Hz, 3H) ppm.

2,7-Diethyl-1,8-naphthyridine (**B**). According to a reported procedure, with minor modifications.^3^ 2-Ethyl-1,8-naphthyridine (**A**) (6.44 g, 0.040 mol) was dissolved in dry toluene (250 mL) and placed in a flame-dried 1000 mL three-neck round bottom flask equipped with a dropping funnel under an argon atmosphere. The yellow solution was cooled to approximately -20 °C, and ethyllithium (0.162 mol, 4.0 equiv., 0.54 M solution in dry Et_2_O) was slowly added via canula at this temperature. The solution mixture was then allowed to warm to room temperature and stirred for 12 h. Then the reaction mixture was quenched with H_2_O (200.0 mL) and extracted with Et_2_O 3 times. The combined organic layers were dried over Na_2_SO_4_, filtrated, and concentrated under reduced pressure to produce the intermediate 2,7-diethyl-1,2-dihydro-1,8-naphthyridine as an orange oil. This intermediate was dissolved in a saturated solution of KMnO_4_ in acetone (700 mL) and stirred for 16 h. The mixture was filtered through a plug of silica and concentrated under reduced pressure to yield 2,7-diethyl-1,8-naphthyridine (**B**) (78% yield). ^1^H NMR (400.13 MHz, Chloroform-d): *δ =* 8.03 (d, *J* = 8.3 Hz, 1H), 7.32 (d, *J* = 8.3 Hz, 1H), 3.05 (q, *J* = 7.6 Hz, 4H), 1.42 (t, *J* = 7.6 Hz, 6H) ppm.

1,1′-(1,8-Naphthyridine-2,7-diyl)diethanone (**C**). According to a reported procedure, with minor modifications.^3^ 2,7-Diethyl-1,8-naphthyridine (**B**) (1.1 g, 5.90 mmol) and tert-butyl hydroperoxide (*ca.* 5.5 M in hexane) (40.8 mL, 224.64 mmol, 48 eq.) were charged in a pressure tube. The yellow solution was allowed to stir at 140 °C for 18 h. The reaction solution was then cooled down to room temperature and then H_2_O (50 mL) and EtOAc (50 mL) were added. Subsequently, the aqueous layer was extracted with EtOAc 3 times. The combined organic layers were dried over Na_2_SO_4_, filtrated, and concentrated under reduced pressure. Then the crude product was further purified by column chromatography (SiO_2_, 40% EtOAc in pentane) to afford compound **C** (40% yield). ^1^H NMR (400.13 MHz, Chloroform-d): *δ =* 8.40 (d, *J* = 8.4 Hz, 2H), 8.31 (d, *J* = 8.4 Hz, 2H), 2.97 (s, 6H) ppm.

SBA-15 was synthesized following a previously reported procedure.^4, 5^ Pluronic P123 (11.744 g) was dissolved in deionized water (303.411 g) with concentrated HCl (69.164 g) in a polypropylene (PP) bottle (Nalgene/Thermo Fisher Scientific) under controlled temperature (40 °C) and stirred at 500 rpm for 4 h using triangular-prismatic stirring bar (length *ca.* 5 cm). Tetraethyl orthosilicate (TEOS, 25.019 g) was then added to the solution under continuous stirring (500 rpm) for 5 min. The reaction mixture was kept under static conditions at 40 °C for 24 h, followed by hydrothermal treatment at 100 °C for another 24 h. The resulting solid was collected by suction filtration, washed with deionized water, and dried overnight in air at room temperature to yield the as-synthesized materials (6.394 g) as a fine white powder. The surfactant was removed by calcination at 540 °C for 6 h using a controlled heating program. The calcined SBA-15 was subsequently activated under vacuum at 250 °C for 4 h and stored in a glovebox under an argon atmosphere. Nitrogen content (elemental analysis) 0.01 %. *a*_BET_ 780 m^2^ g^-1^ , *V*_pore_ 1.04 cm^3^ g^-1^, and *d*_pore_ 7.7 nm. DRIFTS: ṽ = 3746 (m, Si-OH), 1172 (s), 1072 (s), 814 (m), 450 (m) cm^-1^.

**Grafting Reactions**

**APTES@SBA-15_250_**. SBA-15_250_ (500.5 mg, 1.183 mmol of SiOH) was suspended in toluene, and 3-aminopropyltriethoxysilane (APTES) (392.8 mg, 1.774 mmol) in toluene was added. The suspension was continuously stirred at 80 °C for 18 h. Subsequently, the solid material was collected by centrifugation and washed three times with toluene and twice with hexane. The residual solvent was removed under vacuum to afford APTES@SBA-15_250_ (627.7 mg) as a white powder. Elemental analysis found: C 11.51, H 2.34, N 1.88. N_2_ physisorption: *a*_BET_ = 399 m^2^ g^−1^, *V*_pore_ = 0.59 cm^3^ g^−1^, and *d*_pore_ = 6.6 nm. DRIFTS: ṽ = 3368, 3319, 2979, 2932, 2893, 2873, 1875, 1593, 1446, 1392 cm^−1^.

**APTMS@SBA-15_250_**. SBA-15_250_ (404.1 mg, 0.955 mmol of SiOH) was suspended in toluene, and 3-aminopropyltrimethoxysilane (APTMS) (256.9 mg, 1.433 mmol) in toluene was added. The suspension was continuously stirred at 80 °C for 18 h. Subsequently, the solid material was collected by centrifugation and washed three times with toluene and twice with hexane. The residual solvent was removed under vacuum to afford **APTMS@SBA-15_250_** (507.2 mg) as a white powder. Elemental analysis found: C 9.35, H 2.19, N 1.97. N_2_ physisorption: *a*_BET_ = 464 m^2^ g^−1^, *V*_pore_ = 0.67 cm^3^ g^−1^, and *d*_pore_ = 6.4 nm. DRIFTS: ṽ = 3406, 2979, 2934, 2902, 2882, 1858, 1595, 1449, 1392 cm^−1^.

**NDI-APTES@SBA-15_250_. APTES@SBA-15_250_** (500.2 mg, 0.670 mmol of N) was suspended in ethanol and compound **C** (86.12 mg, 0.400 mmol) was added. The suspension was stirred at 110 °C for 72 h, then the solid component was collected by centrifugation, and washed three times with ethanol and two times with hexane until the color of supernatant became colorless. Then the residual solvent was removed under reduced pressure to yield **NDI-APTES@SBA-15_250_** (460.4 mg) as an orange powder. Elemental analysis found: C 17.20, H 2.43, N 2.78. N_2_ physisorption: *a*_BET_ = 380 m^2^ g^−1^, *V*_pore_ = 0.49 cm^3^ g^−1^, and *d*_pore_ = 5.8 nm. DRIFTS: ṽ = 2977, 2933, 2893, 1893, 1638, 1600, 1501, 1448, 1394, 1370 cm^−1^.

**NDI-APTMS@SBA-15_250_. APTMS@SBA-15_250_** (401.5 mg, 0.566 mmol of N) was suspended in ethanol and compound **C** (72.76 mg, 0.340 mmol) was added. The suspension was stirred at 110 °C for 72 h, then the solid component was collected by centrifugation, and washed three times with ethanol until the color of supernatant became colorless and two times with hexane. Then the residual solvent was removed under reduced pressure to yield **NDI-APTMS@SBA-15_250_** (380.1 mg) as an orange powder. Elemental analysis found: C 15.46, H 2.34, N 2.61. N_2_ physisorption: *a*_BET_ = 413 m^2^ g^−1^, *V*_pore_ = 0.55 cm^3^ g^−1^, and *d*_pore_ = 6.3 nm. DRIFTS: ṽ = 2979, 2934, 2895, 1876, 1641, 1602, 1502, 1444, 1395, 1368 cm^−1^.

**Ni-NDI-APTES@SBA-15_250_.** **NDI-APTES@SBA-15_250_** (405.6 mg, 0.807 mmol of N) was suspended in C_6_H_6_ and Ni(COD)_2_ (222.0 mg, 0.807 mmol) was added. The suspension was stirred at ambient temperature for 48 h. Then the solid component was collected by centrifugation, and washed three times with C_6_H_6_ until the color of supernatant became colorless and two times with hexane. Then the residual solvent was removed under reduced pressure to yield **Ni-NDI-APTES@SBA-15_250_** (386.7 mg) as a dark green powder. Elemental analysis found: C 18.11, H 2.57, N 2.61. N_2_ physisorption: *a*_BET_ = 333 m^2^ g^−1^, *V*_pore_ = 0.43 cm^3^ g^−1^, and *d*_pore_ = 5.6 nm. DRIFTS: ṽ = 2977, 2930, 2895, 1859, 1633, 1598, 1517, 1441, 1392, 1373 cm^−1^.

**Ni-NDI-APTMS@SBA-15_250_.** **NDI-APTMS@SBA-15_250_** (300.8 mg, 0.559 mmol of N) was suspended in C_6_H_6_ and Ni(COD)_2_ (153.9 mg, 0.559 mmol) was added. The suspension was stirred at ambient temperature for 48 h. Then the solid component was collected by centrifugation, and washed three times with C_6_H_6_ until the color of supernatant became colorless and two times with hexane. Then the residual solvent was removed under reduced pressure to yield **Ni-NDI-APTMS@SBA-15_250_** (281.3 mg) as a dark green powder. Elemental analysis found: C 17.06, H 2.16, N 2.51. N_2_ physisorption: *a*_BET_ = 357 m^2^ g^−1^, *V*_pore_ = 0.47 cm^3^ g^−1^, and *d*_pore_ = 6.2 nm. DRIFTS: ṽ = 2977, 2932, 2892, 1869, 1640, 1600, 1519, 1445, 1394, 1370 cm^−1^.

**S2. Materials and Compounds Characterization**

Figure S1. Low-angle PXRD patterns of dried support SBA-15.

Low-angle PXRD analysis was performed to confirm the successful synthesis of SBA-15. The diffraction pattern exhibited the characteristic peaks at 2θ = 0.93°, 1.60°, and 1.85°, which can be indexed to the [100], [110] and [200] planes, respectively, characteristic of the ordered 2D-hexagonal symmetry (*p*6*mm*).^4^

Figure S2. SEM a-d) and TEM e-h) of SBA-15.

Figure S3. DRIFT spectra of support material SBA-15_250_ as well as functionalized materials APTMS@SBA-15_250_, NDI-APTMS@SBA-15_250_, and Ni-NDI-APTMS@SBA-15_250_.

Figure S4. a) TGA and b) DTA curves of SBA-15_250_, APTES@SBA-15_250_, NDI-APTES@SBA-15_250_, and Ni-NDI-APTES@SBA-15_250_.

Figure S5. N_2_ physisorption isotherms (a) and pore size distribution (b) of SBA-15_250_, APTMS@SBA-15_250_, NDI-APTMS@SBA-15_250_, and Ni-NDI-APTMS@SBA-15_250_.

**S3. Solid-State NMR Spectroscopy of Grafted Materials**

Figure S6. ^13^C CP/MAS NMR spectra of APTMS@SBA-15_250_ (black) and NDI-APTMS@SBA-15_250_ (red).

Figure S7. Comparison between the ^13^C NMR of the 1,1′-(1,8-Naphthyridine-2,7-diyl)diethanone ligand (black) and ^13^C CP/MAS spectra of NDI-APTES@SBA-15_250_ (red) and NDI-APTMS@SBA-15_250_ (blue).

Figure S8. ^29^Si CP/MAS NMR spectra of APTMS@SBA-15_250_ (black) and NDI-APTMS@SBA-15_250_ (red).

Figure S9. Peak deconvolution and assignment of T^1^, T^2^, and T^3^ species for a) APTES@SBA-15_250_, b) NDI-APTES@SBA-15_250_, c) APTMS@SBA-15_250_ and d) NDI-APTMS@SBA-15_250_.

Table S1. Calibration values of the integrated areas obtained from peak deconvolution for all functionalized materials.

| Samples | T^1^ | T^2^ | T^3^ |
| --- | --- | --- | --- |
| APTES@SBA-15_250_ | 0.33 | 1.00 | 0.10 |
| NDI-APTES@SBA-15_250_ | 0.26 | 1.00 | 0.05 |
| APTMS@SBA-15_250_ | 0.05 | 1.00 | 0.20 |
| NDI-APTMS@SBA-15_250_ | 0.16 | 1.00 | 0.09 |

**S4. XPS (X-ray Photoelectron) Spectroscopy**

Figure S10. a) XPS spectra for (*^i^*^-Pr^NDI)Ni_2_(C_6_H_6_) and Ni-NDI-APTES@SBA-15_250_, XPS peak of Ni 2p for b) (*^i^*^-Pr^NDI)Ni_2_(C_6_H_6_) and c) Ni-NDI-APTES@SBA-15_250_.

**S5. Procedure for the Catalytic Reactions**

Catalytic procedure for phenylacetylene.^6^ The catalyst (3.75 mol% Ni) was charged to a vial. Then phenylacetylene (0.4 mmol) and 1,3,5-trimethylbenzene (0.4 mmol) in C_6_H_6_ (1 mL) were added. The reaction was stirred at 60 °C for 40 min. After finishing the reaction, conversion of substrate was determined by NMR, combined yield of trimers, and the ratio of regioisomers were determined by GC-MS analysis.

Catalytic procedure for ethyl propiolate/methyl propargyl ether.^6^ The catalyst (2 mol% Ni) was charged to a vial. Then ethyl propiolate/methyl propargyl ether (0.4 mmol) and 1,3,5-trimethylbenzene (0.4 mmol) in C_6_H_6_ (0.5 mL) were added. The reaction was stirred at ambient temperature for 11 min. After finishing the reaction, conversion of substrate was determined by NMR, combined yield of trimers, and the ratio of regioisomers were determined by GC-MS analysis.

**S6. Solvent Effect in Cyclotrimerization**

Table S2. Optimizing reaction conditions by varying the solvent.

| Entry | Solvent | Catalyst | Amount of catalyst (mol% Ni) | Conversion (%) | Selectivity (%) | |
| --- | --- | --- | --- | --- | --- | --- |
|  |  |  |  |  | 1,2,4-trimer | 1,3,5-trimer |
| 1 | Benzene | Ni-NDI-APTES@SBA-15_250_ | 2.5 | 84 | 89 | 11 |
| 2 | Toluene | Ni-NDI-APTES@SBA-15_250_ | 2.5 | 76 | 86 | 14 |
| 3 | THF | Ni-NDI-APTES@SBA-15_250_ | 2.5 | 73 | 86 | 14 |
| 4 | Acetonitrile | Ni-NDI-APTES@SBA-15_250_ | 2.5 | 59 | 87 | 13 |

**S7. Catalytic Comparison to Previous Reports**

Table S3. Catalytic comparison between the catalytic reactivity of Ni-NDI-APTES@SBA-15_250_ with previously reported Ni compounds, mononuclear and dinuclear compounds.^6^

| Entry | Substrate | Catalyst | Conversion (%) | Combined yield of trimers (%) | Selectivity (%) | | TON*^a^* |
| --- | --- | --- | --- | --- | --- | --- | --- |
|  |  |  |  |  | 1,2,4-trimer | 1,3,5-trimer |  |
| 1 | -Ph | Ni-NDI-APTES@SBA-15_250_ | >99 | >99 | 81 | 19 | 21.6 |
| 2 | -Ph | (*^i-^*^Pr^NDI)Ni_2_(C_6_H_6_) (**1**) | >99 | >99 | 97 | 3 | 9.7 |
| 3 | -Ph | (*^i-^*^Pr^IP)Ni(COD) (**2**) | 53 | 28 | 85 | 15 | 2.5 |
| 4 | -Ph | (BPY)Ni(COD) (**3**) | 35 | 14 | 78 | 22 | 0.7 |
| 5 | -Ph | (*^i-^*^Pr^DAD)Ni(COD) (**4**) | 13 | 9 | 89 | 11 | 0.2 |
| 6 | -Ph | Ni(COD)_2_ (**5**) | 52 | 23 | 74 | 26 | 1.8 |
| 7 | -Ph | Ni_2_(L)(DPA)*^b^* (**6**) | 64 | 64 | 59 | 5 | 2.4 |
| 8 | -CO_2_Et | Ni-NDI-APTES@SBA-15_250_ | 63 | 63 | 72 | 28 | 28.6 |
| 9 | -CO_2_Et | Ni-NDI-APTES@SBA-15_250_*^c^* | 97 | 97 | 79 | 21 | 37.2 |
| 10 | -CO_2_Et | (NDI)Ni_2_(C_6_H_6_) (**1**) | >99 | 90 | 76 | 24 | 34.2 |
| 11 | -CO_2_Et | (*^i-^*^Pr^IP)Ni(COD) (**2**) | 33 | 8 | 74 | 26 | 2.0 |
| 12 | -CO_2_Et | (BPY)Ni(COD) (**3**) | 29 | 12 | 85 | 15 | 3.0 |
| 13 | -CO_2_Et | (*^i-^*^Pr^DAD)Ni(COD) (**4**) | 72 | 15 | 76 | 24 | 8.2 |
| 14 | -CO_2_Et | Ni(COD)_2_ (**5**) | 93 | 14 | 75 | 25 | 10.0 |
| 15 | -CO_2_Et | Ni_2_(L)(DPA)*^b^* (**6**) | 46 | 33 | 28 | 7 | 2.1 |
| 16 | -CH_2_OMe | Ni-NDI-APTES@SBA-15_250_ | 55 | 55 | 76 | 24 | 23.0 |
| 17 | -CH_2_OMe | Ni-NDI-APTES@SBA-15_250_*^d^* | 98 | 98 | 75 | 25 | 36.0 |
| 18 | -CH_2_OMe | (*^i-^*^Pr^NDI)Ni_2_(C_6_H_6_) (**1**) | >99 | >99 | 99 | 1 | 49.0 |
| 19 | -CH_2_OMe | (*^i-^*^Pr^IP)Ni(COD) (**2**) | 2 | <1 | 50 | 50 | 1.0 |
| 20 | -CH_2_OMe | (BPY)Ni(COD) (**3**) | 9 | 1 | 55 | 45 | 5.0 |
| 21 | -CH_2_OMe | (*^i-^*^Pr^DAD)Ni(COD) (**4**) | 2 | <1 | 74 | 26 | 1.5 |
| 22 | -CH_2_OMe | Ni(COD)_2_ (**5**) | 23 | 2 | 55 | 45 | 0.3 |
| 23 | -CH_2_OMe | Ni_2_(L)(DPA)*^b^* (**6**) | 27 | 14 | 10 | 4 | 0.2 |

*^a^*TON: mol of 1,2,4-trimeric product per mol catalyst used. *^b^*Ni_2_(L)(DPA) = Ni_2_(*N,N’-*(2,7-di-*tert*-butyl-9,9-dimethyl-9*H*-xanthene-4,5-diyl)bis(1-(pyridine-2-yl)methanimine))(DPA) *^c^*Using 2 mol% Ni. *^d^*The reaction was carried out for 60 min, using 2 mol% Ni.

Table S4. Substrate scope of the heterogeneous Ni-NDI-APTES@SBA-15_250_ catalyst in comparison with the previously reported homogeneous dinuclear nickel (**1**) catalyst.

| Entry | Substrate | Catalyst | Condition | Conversion (%) | Selectivity (%) | | | |
| --- | --- | --- | --- | --- | --- | --- | --- | --- |
|  |  |  |  |  | 1,2,4 | 1,3,5 | | COTs |
| 1*^a^* | **I** | (*^i-^*^Pr^NDI)Ni_2_(C_6_H_6_) (**1**) | 10 mol% Ni, 60 °C, 40 min | >99 | 80 | 20 | | - |
| 2 | **I** | Ni-NDI-APTES@SBA-15_250_ | 5 mol% Ni, 60 °C, 40 min | >99 | 97 | 3 | | - |
| 3 | **II** | (*^i-^*^Pr^NDI)Ni_2_(C_6_H_6_) (**1**) | 2 mol% Ni, rt, 120 min | 29 | 100 | - | | - |
| 4 | **II** | Ni-NDI-APTES@SBA-15_250_ | 2 mol% Ni, rt, 120 min | 49 | 92 | 8 | | - |
| 5 | **III** | (*^i-^*^Pr^NDI)Ni_2_(C_6_H_6_) (**1**) | 2 mol% Ni, rt, 120 min | 71 | 57 | 43 | | - |
| 6 | **III** | Ni-NDI-APTES@SBA-15_250_ | 2 mol% Ni, rt, 120 min | 78 | 100 | - | | - |
| 7 | **IV** | (*^i-^*^Pr^NDI)Ni_2_(C_6_H_6_) (**1**) | 2 mol% Ni, rt, 120 min | 9 | 100 | - | | - |
| 8 | **IV** | Ni-NDI-APTES@SBA-15_250_ | 2 mol% Ni, rt, 120 min | <1 | *n.d.* | *n.d.* | | *n.d.* |
| 9*^a^* | **V** | (*^i-^*^Pr^NDI)Ni_2_(C_6_H_6_) (**1**) | 2 mol% Ni, rt, 11 min | 99 | 68 | 22 | | 10 |
| 10 | **V** | Ni-NDI-APTES@SBA-15_250_ | 2 mol% Ni, rt, 11 min | 97 | 79 | 21 | | - |
| 11*^a^* | **VI** | (*^i-^*^Pr^NDI)Ni_2_(C_6_H_6_) (**1**) | 2 mol% Ni, rt, 11 min | >99 | 99 | 1 | | - |
| 12 | **VI** | Ni-NDI-APTES@SBA-15_250_ | 2 mol% Ni, rt, 11 min | 64 | 76 | 24 | | - |
| 13 | **VII** | (*^i-^*^Pr^NDI)Ni_2_(C_6_H_6_) (**1**) | 3.75 mol% Ni, rt, 60 min | 70 | 79 | 21 | | - |
| 14 | **VII** | Ni-NDI-APTES@SBA-15_250_ | 3.75 mol% Ni, rt, 60 min | 54 | 82 | 18 | | - |
| 15 | **VIII** | (*^i-^*^Pr^NDI)Ni_2_(C_6_H_6_) (**1**) | 3.75 mol% Ni, 60 °C, 60 min | 98 | 92 | 8 | | - |
| 16 | **VIII** | Ni-NDI-APTES@SBA-15_250_ | 3.75 mol% Ni, 60 °C, 60 min | 95 | 92 | 8 | | - |
| Entry | Substrate | Catalyst | Condition | Conversion (%) | Selectivity (Yield %) | | | |
|  |  |  |  |  | **IXa, Xa** | | **IXb, Xb** | |
| 17*^a^* | **IX** | (*^i-^*^Pr^NDI)Ni_2_(C_6_H_6_) (**1**) | 2 mol% Ni, rt, 15 min | - | (94) | | - | |
| 18 | **IX** | Ni-NDI-APTES@SBA-15_250_ | 2 mol% Ni, rt, 15 min | 46 | 5 (2) | | 95 (44) | |
| 19*^a^* | **X** | (*^i-^*^Pr^NDI)Ni_2_(C_6_H_6_) (**1**) | 2 mol% Ni, rt, 15 min | - | (84) | | - | |
| 20 | **X** | Ni-NDI-APTES@SBA-15_250_ | 2 mol% Ni, rt, 15 min | 53 | <1 (<1) | | >99 (53) | |

*^a^*Catalytic data for the (*^i-^*^Pr^NDI)Ni_2_(C_6_H_6_) (**1**) from literature.^6^

**S8. Reusability Study**

Table S5. Recycling performance of Ni-NDI-APTES@SBA-15_250_ over four consecutive catalytic cycles.

| Number of cycles | Conversion (%) | Selectivity of trimeric products (%) | Ratio of 1,2,4 to 1,3,5 |
| --- | --- | --- | --- |
| 1 | 76 | 100 | 79:21 |
| 2 | 72 | 100 | 77:23 |
| 3 | 61 | 100 | 79:21 |
| 4 | 49 | 100 | 79:21 |

Figure S11. Filtration test for Ni-NDI-APTES@SBA-15_250_; the catalyst was removed by filtration after 20 min, and the obtained homogeneous solution was allowed to react for an additional 20 min.

Table S6. Results of filtration test for Ni-NDI-APTES@SBA-15_250_.

| Time (min) | Conversion (%) |
| --- | --- |
| 0 | 0 |
| 20 | 76 |
| 40 | 76 |

Figure S12. Low-angle PXRD patterns of Ni-NDI-APTES@SBA-15_250_ (Fresh) and Ni-NDI-APTES@SBA-15_250_ (Reused).

Table S7. Textural properties comparison between fresh and reused materials

| Material | Physical parameters | | |
| --- | --- | --- | --- |
|  | *a*_BET_ (m^2^ g^-1^) | *V*_pore_ (cm^3^ g^-1^_)_ | *d*_pore_ (nm)*^c^* |
| Ni-NDI-APTES@SBA-15_250_ (Fresh) | 333 | 0.43 | 5.6 |
| Ni-NDI-APTES@SBA-15_250_ (Reused) | 277 | 0.37 | 5.4 |

Figure S13. N_2_ physisorption isotherms (a) and pore size distribution (b) of Ni-NDI-APTES@SBA-15_250_ (Fresh) and Ni-NDI-APTES@SBA-15_250_ (Reused).

Figure S14. DRIFT spectra comparison between Ni-NDI-APTES@SBA-15_250_ (Fresh) and Ni-NDI-APTES@SBA-15_250_ (Reused).

Figure S15. SEM (top) and TEM (bottom) of fresh Ni-NDI-APTES@SBA-15_250_.

Figure S16. SEM (top) and TEM (bottom) of reused Ni-NDI-APTES@SBA-15_250_.

Figure S17. HRTEM of fresh Ni-NDI-APTES@SBA-15_250_.

Figure S18. HRTEM of reused Ni-NDI-APTES@SBA-15_250_.

Figure S19. HAADF-STEM and STEM-EDX of fresh Ni-NDI-APTES@SBA-15_250_.

Figure S20. HAADF-STEM and STEM-EDX of reused Ni-NDI-APTES@SBA-15_250_.

**S9. NMR Spectra of Crude Mixtures from Representative Catalytic Reactions**

Figure S21. Crude ^1^H NMR spectrum (CDCl_3_, 400.13 MHz, 26 °C) of cyclotrimerization of phenylacetylene using Ni-NDI-APTES@SBA-15_250_ as a catalyst, and TMB (1,3,5-trimethylbenzene) as an internal standard (Table 2, entry 3).

Figure S22. Crude ^1^H NMR spectrum (C_6_D_6_, 400.13 MHz, 26 °C) of cyclotrimerization of ethyl propiolate using Ni-NDI-APTES@SBA-15_250_ as a catalyst, and TMB (1,3,5-trimethylbenzene) as an internal standard (Table 2, entry 10).

Figure S23. Crude ^1^H NMR spectrum (C_6_D_6_, 400.13 MHz, 26 °C) of cyclotrimerization of methyl propargyl ether using Ni-NDI-APTES@SBA-15_250_ as a catalyst, and TMB (1,3,5-trimethylbenzene) as an internal standard (Table 2, entry 14).

Figure S24. Crude ^1^H NMR spectrum (C_6_D_6_, 400.13 MHz, 26 °C) of cyclotrimerization of 1,6-heptadiyne using Ni-NDI-APTES@SBA-15_250_ as a catalyst, and TMB (1,3,5-trimethylbenzene) as an internal standard compared with the 1,6-heptadiyne spectrum.

Figure S25. Crude ^1^H NMR spectrum (C_6_D_6_, 400.13 MHz, 26 °C) of cyclotrimerization of dipropargyl ether using Ni-NDI-APTES@SBA-15_250_ as a catalyst, and TMB (1,3,5-trimethylbenzene) as an internal standard compared with the dipropargyl ether spectrum.

**S10. GC Chromatograms of Crude Mixtures from Representative Catalytic Reactions**

Figure S26. Crude GC Chromatogram of cyclotrimerization of phenylacetylene using Ni-NDI-APTES@SBA-15_250_ as a catalyst (Table 2, entry 3).

Figure S27. Crude GC Chromatogram of cyclotrimerization of ethyl propiolate using Ni-NDI-APTES@SBA-15_250_ as a catalyst (Table 2, entry 10).

Figure S28. Crude GC Chromatogram of cyclotrimerization of methyl propargyl ether using Ni-NDI-APTES@SBA-15_250_ as a catalyst (Table 2, entry 14).

Figure S29. Crude GC Chromatogram of cyclotrimerization of 1,6-heptadiyne using Ni-NDI-APTES@SBA-15_250_ as a catalyst.

Figure S30. Crude GC Chromatogram of cyclotrimerization of dipropargyl ether using Ni-NDI-APTES@SBA-15_250_ as a catalyst.

**References**

(1) Grahlow, F.; Beitlberger, J.; Martin, M.; Juriatti, E.; Peisert, H.; Scheele, M.; Ströbele, M.; Romao, C. P.; Meyer, H.-J. Structural modifications of M5O4I11 (M = Nb, Ta) cluster networks from heterogeneous solid-state reactions. *Dalton Transactions* **2025**, *54* (44), 16593-16604, 10.1039/D5DT02097B.

(2) Dormer, P. G.; Eng, K. K.; Farr, R. N.; Humphrey, G. R.; McWilliams, J. C.; Reider, P. J.; Sager, J. W.; Volante, R. P. Highly Regioselective Friedländer Annulations with Unmodified Ketones Employing Novel Amine Catalysts:  Syntheses of 2-Substituted Quinolines, 1,8-Naphthyridines, and Related Heterocycles. *The Journal of Organic Chemistry* **2003**, *68* (2), 467-477.

(3) Braconi, E.; Cramer, N. A Chiral Naphthyridine Diimine Ligand Enables Nickel-Catalyzed Asymmetric Alkylidenecyclopropanations. *Angewandte Chemie International Edition* **2020**, *59* (38), 16425-16429.

(4) Poolwong, J.; Kracht, F.; Moinet, E.; Liang, Y.; D’Elia, V.; Anwander, R. Samarium- and Ytterbium-Grafted Periodic Mesoporous Silica for Carbon Dioxide Capture and Conversion. *Inorganic Chemistry* **2023**, *62* (43), 17972-17984.

(5) Lee, H. I.; Kim, J. H.; Stucky, G. D.; Shi, Y.; Pak, C.; Kim, J. M. Morphology-selective synthesis of mesoporous SBA-15 particles over micrometer, submicrometer and nanometer scales. *Journal of Materials Chemistry* **2010**, *20* (39), 8483-8487, 10.1039/C0JM00820F.

(6) Pal, S.; Uyeda, C. Evaluating the Effect of Catalyst Nuclearity in Ni-Catalyzed Alkyne Cyclotrimerizations. *Journal of the American Chemical Society* **2015**, *137* (25), 8042-8045.
